# Development and characterization of an animal model of Japanese encephalitis virus infection in adolescent C57BL/6 mouse

**DOI:** 10.1242/dmm.049176

**Published:** 2021-10-22

**Authors:** Aarti Tripathi, Arup Banerjee, Sudhanshu Vrati

**Affiliations:** 1 Infection and Immunology, Translational Health Science and Technology Institute, Faridabad 121001, India; 2 Laboratory of Virology, Regional Centre for Biotechnology, Faridabad 121001, India

**Keywords:** C57BL/6, JEV mouse model, Virulence, Proteome profile

## Abstract

A mouse-adapted isolate of Japanese encephalitis virus (JEV), designated as JEV-S3, was generated by serially passaging the P20778 strain of the virus in 3- to 4-week-old C57BL/6 mice. Blood-brain barrier leakage was evident in JEV-S3-infected mice, in which viral antigens and RNA were consistently demonstrated in the brain, along with infiltration of activated immune cells, as evidenced by an increased CD45^+^CD11b^+^ cell population. Histopathology studies showed the presence of perivascular cuffing, haemorrhage and necrotic foci in the virus-infected brain, conforming to the pathological changes seen in the brain of JEV-infected patients. Mass spectrometry studies characterized the molecular events leading to brain inflammation in the infected mice. Notably, a significant induction of inflammatory cytokines, such as IFNγ, IL6, TNFα and TGFβ, was observed. Further, genome sequencing of the JEV-S3 isolate identified the mutations selected during the mouse passage of the virus. Overall, we present an in-depth characterization of a robust and reproducible mouse model of JEV infection. The JEV-S3 isolate will be a useful tool to screen antivirals and study virus pathogenesis in the adolescent mouse model.

## Abstract (precis)

**Summary:** We generated a mouse-adapted Japanese encephalitis virus (JEV) isolate to develop and characterize a mouse model of JEV infection, which could be a useful tool to screen antivirals and study JEV pathogenesis.

## INTRODUCTION

Japanese encephalitis virus (JEV) is a member of the Flaviviridae family of animal viruses. The virus is enveloped, having a single strand of the positive-sense RNA genome of ∼11 kb. JEV is responsible for most cases of viral encephalitis in Asia, where ∼70,000 cases and ∼15,000 JEV-related deaths are reported each year from the epidemics of Japanese encephalitis (JE). JEV is transmitted by mosquitoes, primarily *Culex tritaeniorhynchus*, in a natural life cycle involving wild birds and pigs serving as maintenance host and amplifying host, respectively. After being bitten and infected by mosquitoes, humans are essentially the dead-end hosts for the virus. JE in humans is manifested clinically as altered consciousness, muscle rigidity, cranial nerve palsies, abnormal movements and seizures. JEV antigens and RNA are localized mainly in neurons, suggesting that neuronal viral cytolysis is important for pathogenesis (Fu et al., 2019). Neuroinflammation is the hallmark of the virus infection, causing massive neuronal damage that may result in a permanent neurological sequel. A tissue culture JE vaccine has become available in recent times, although no virus-specific therapeutics are available. Continuous efforts have been made to identify novel antivirals against JEV, and several drug molecules have been tested in small-animal models (Shi et al., 2014; Wei et al., 2020; Ye et al., 2014). However, these animal models show varying susceptibility. In general, older mice (>4 weeks old) are less susceptible than neonates (Ye et al., 2014). The route of inoculation also determines the susceptibility of the animals (Chai et al., 2019). Moreover, different mouse strains exhibited different susceptibility to JEV infection (Hsieh et al., 2019; Miura et al., 1988; Yun et al., 2016). Most researchers have used BALB/c mice (Kulkarni et al., 2012; Ye et al., 2014; Zhu et al., 2015) for studying JEV infection as C57BL/6 mice were reported to be less susceptible to JEV infection (Miura et al., 1988).

C57BL/6 is a common inbred mouse strain used in modern biomedical research, and knockout mice in the C57BL/6 background are widely used to study viral pathogenesis. Owing to the lack of a JEV isolate capable of producing clinical disease in C57BL/6 mice, it is challenging to study JEV pathogenesis in knockout mice with C57BL/6 background. We have established and characterized a mouse model for JEV infection via intraperitoneal (IP) inoculation of the virus in 3- to 4-week-old C57BL/6 mice. The mouse-adapted Stage-3 (S3) JEV (JEV-S3) was isolated by the sequential passage of the P20778 strain of the virus in neonatal and adolescent mice. The JEV-S3-infected adolescent C57BL/6 and BALB/c mice consistently developed clinical signs of infection similar to those seen in humans, such as weight loss, hunchback posture, tremors and, occasionally, hindlimb paralysis. Viral antigens and RNA were consistently demonstrated in the mouse brain. Quantitative mass spectrometry studies were carried out to characterize the molecular events in the brain tissue caused by JEV infection in the C57BL/6 mouse model. The expression of several interferon-stimulated genes was found to be highly upregulated in the JEV-infected mouse brain. Notably, a significant induction of inflammatory cytokines, such as IFNγ, IL6, TNFα (also known as TNF) and TGFβ was observed. Further, genome sequencing of the mouse-adapted JEV was performed to identify the mutations selected during the passage of the virus in the mouse. In the end, we show the utility of this mouse model of JEV infection for testing the potential antiviral molecules.

## RESULTS

### Adaptation of P20778 strain of JEV to C57BL/6 mice

**Fig. 1.**
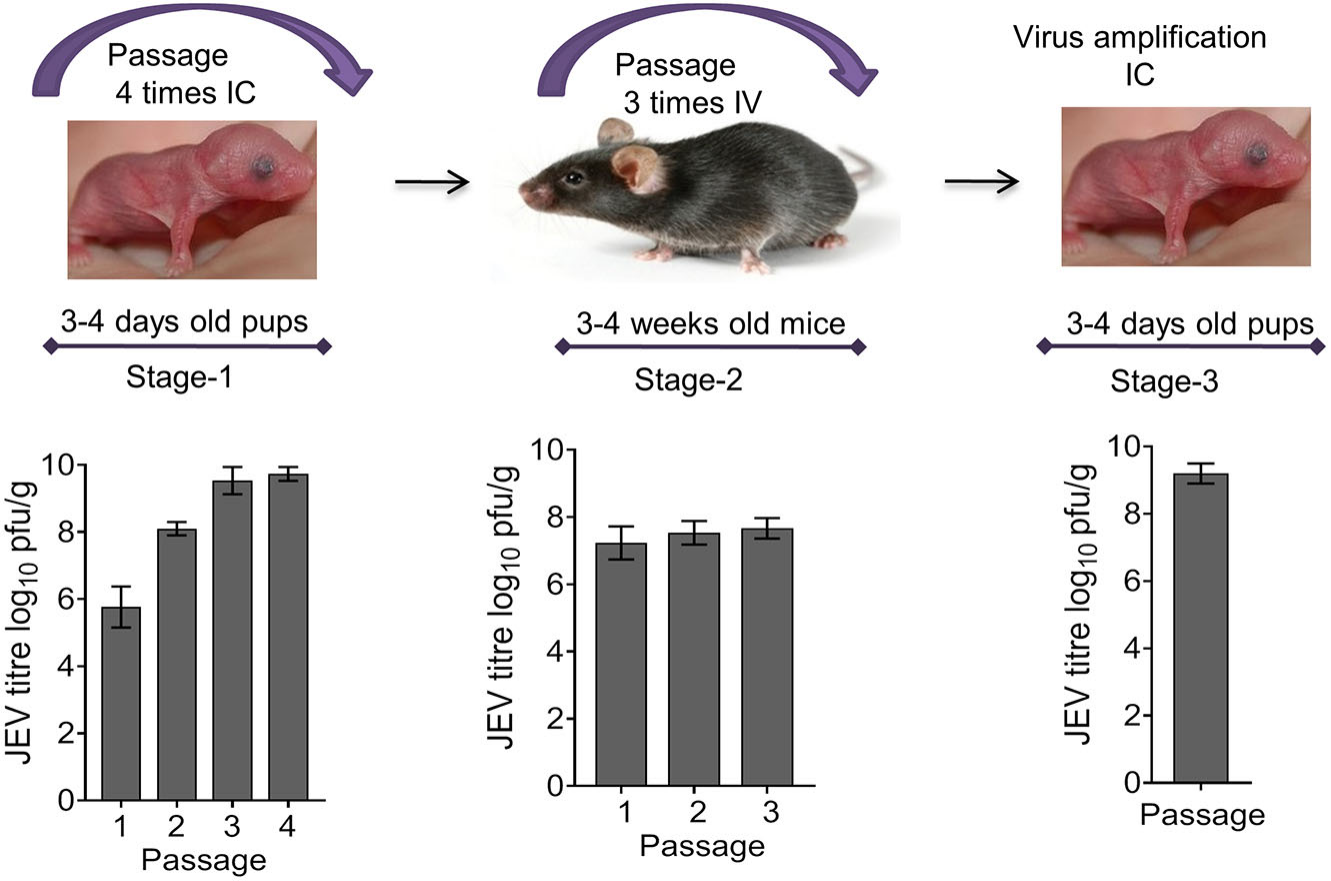
**Generation of mouse-adapted Japanese encephalitis virus (JEV).** The porcine kidney (PS) cell-grown, sucrose gradient-purified JEV (P20078 strain) was sequentially passaged through intracerebral (IC) inoculation in C57BL/6 pups (3-4 days old, *n*=6 in each passage) (Stage-1) followed by sequential passaging in adolescent mice (3-4 weeks old) through the intravenous (IV) route (Stage-2). The virus was further amplified in the brains of pups inoculated through the IC route. Top: schematic of the mouse adaption process. Bottom: JEV titres in the brain tissue of mice at each passage level.

Although the neonatal C57BL/6 mice are susceptible to JEV infection, adolescent mice are resistant. To generate a JEV isolate that can reproducibly infect C57BL/6 mice and produce clinical signs, we adapted the cell culture-grown virus (parent) to C57BL/6 pups then to the adolescent mice. A schematic of the virus adaptation process used is presented in Fig. 1. The cell culture-grown JEV was passaged four times in mouse pups through the intracerebral (IC) route [Stage-1 (S1)]. On subsequent passaging, the virus titre in the mouse brain increased from ∼10^5^ plaque-forming units (pfu)/g (passage 1) to ∼10^9^ pfu/g (passage 3 and 4) (Fig. 1). The S1 virus (passage 4) was used to infect adolescent (3-4 weeks old) C57BL/6 mice through the intravenous (IV) route. A few of the virus-inoculated mice developed clinical signs of the disease, such as abnormal gait, whole-body tremor and limb paralysis. On day 12-15 post-infection (pi), brain tissue from mice showing disease symptoms was harvested and virus suspension prepared. This harvest was used to passage the virus two more times in adolescent mice through the IV route [Stage-2 (S2)]. During S2 of passaging, the virus titre remained ∼10^8^ pfu/g of brain tissue. The S2 virus was further amplified in the pups’ brain through the IC route, yielding a titre of ∼5×10^8^ pfu/g of brain tissue (S3).

### Virulence of mouse-adapted JEV in C57BL/6 mice

**Fig. 2.**
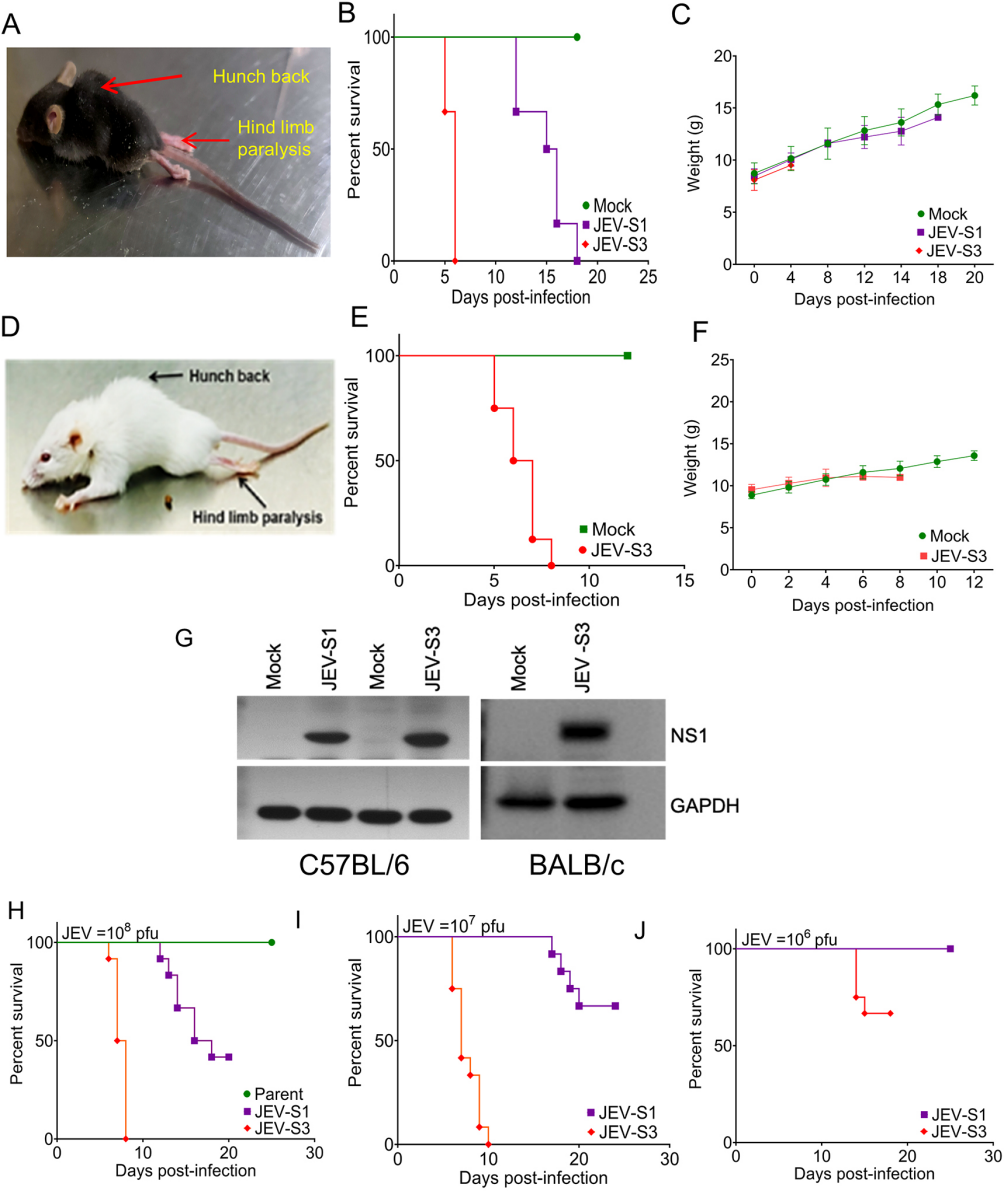
**Lethality of mouse-adapted JEV in C57BL/6 and BALB/c mice inoculated IV.** C57BL/6 and BALB/c mice (3-4 weeks old) were injected with 100 µl JEV [10^8^ plaque-forming units (pfu)] through the tail vein at Stage-1 (S1) or Stage-3 (S3) of mouse adaption (six mice in each group) and assessed for Japanese encephalitis (JE) clinical symptoms and body weight post-infection. (A,D) Images depicting symptoms in JEV-infected C57BL/6 (A) and BALB/c (D) mice. JEV-S3-infected mice show a hunchback posture, fur ruffling and hindlimb paralysis. (B,E) Kaplan–Meier survival curves of JEV-infected C57BL/6 (B) and BALB/c (E) mice. (C,F) The change in body weight of JEV-infected C57BL/6 (C) and BALB/c (F) mice. (G) C57BL/6 and BALB/c mice (3-4 weeks old) were inoculated intraperitoneally (IP) with mock or JEV. The brain tissue was collected 24 h after the appearance of clinical symptoms and protein lysate prepared. The abundance of JEV NS1 protein in the brain of virus-infected mice is shown. GAPDH was used as a loading control. (H-J) C57BL/6 mice (3-4 weeks old, *n*=12 mice in each group) were inoculated IP with 100 µl JEV-S1 or JEV-S3 [10^8^ pfu (H), 10^7^ pfu (I), 10^6^ pfu (J)] and scored for the JE clinical symptoms and body weight each day. Kaplan–Meier survival curves of animals infected with different doses of viruses using a log-rank test are shown.

To assess the virulence of the passaged virus at different stages and the resulting susceptibility of mice, 3- to 4-week-old C57BL/6 mice were injected through the tail vein with 10^8^ pfu of JEV and monitored for 25 days or until death (Fig. 2A-C). Inoculation of the tissue-cultured parent virus caused no signs of sickness in mice, and they continued to gain weight. The median survival time (MST) for these mice was >25 days. All the mice inoculated with S1 JEV (JEV-S1) died by day 18 pi. The virus-infected mice exhibited mostly body stiffing and piloerection (score, 2). The MST for this group of mice was 17 days, and mice generally survived for 2-3 days after the development of symptoms. Compared to the JEV-S1 virus-infected mice, mice infected with JEV-S3 showed an early drop in body weight, and these mice showed higher susceptibility to the virus. All mice in this group died by 8 days pi, with an MST of 7.5 days. The JEV-S3-infected mice developed body stiffening, piloerection, restriction of movement and hind limb paralysis (score, 3-4), and mice died within a day of the onset of symptoms.

The lethality of the graded doses of the mouse-adapted virus was then checked in 3- to 4-week-old C57BL/6 mice inoculated IP (Fig. 2H-J). The onset of morbidity and mortality in virus-infected mice occurred in a dose-dependent manner. The parent virus tested at the highest dose (10^8^ pfu) resulted in no clinical symptoms and no mortality in mice. JEV-S3 began to produce the clinical signs, including mortality, in mice earlier than demonstrated by the JEV-S1 virus at all doses tested. The MST for the mice inoculated with 10^8^ pfu of JEV-S1 and JEV-S3 was 17 and 7.5 days, respectively (Fig. 2H). For the 10^7^ pfu dose, the MST for JEV-S1 and-S3 was <25 and 7 days, respectively. For the 10^6^ pfu dose, the JEV-S1 virus did not cause any mortality, whereas the JEV-S3 virus caused only ∼30% mortality (Fig. 2I,J). The JEV-S3-infected mice showed a loss of weight, both in the 10^8^ and 10^7^ pfu dose groups (Fig. S4).

These observations suggest that JEV-S3 was more virulent than JEV-S1, and JEV-S3 caused noticeable disease symptoms in mice injected IV and IP. We observed that mice injected with JEV-S3 through the IP or IV route developed JE clinical symptoms within 5-6 days pi, and the MST was similar. Whereas JEV-S3-infected young adolescent mice of 3-4 weeks of age consistently developed disease symptoms including mortality, older mice of 6-8 weeks age developed only a few mild symptoms and they all recovered from the infection. Notably, 3- to 4-week-old BALB/c mice were equally susceptible to infection with JEV-S3 given IP (Fig. 2D-F). Viral protein NS1 was detected in the brain tissue of all the infected mice (Fig. 2G).

To quantify virus virulence, the median lethal dose (LD_50_) of the viruses was determined in 3- to 4-week-old C57BL/6 mice. The LD_50_ of JEV-S3 was 10^6.25^ pfu, which was lower than the LD_50_ of 10^8.38^ pfu for JEV-S1, thus further confirming that JEV-S3 was more virulent than JEV-S1 in 3- to 4-week-old C57BL/6 mice.

### JEV-S3 infection causes breach of the blood-brain barrier (BBB) in mice

**Fig. 3.**
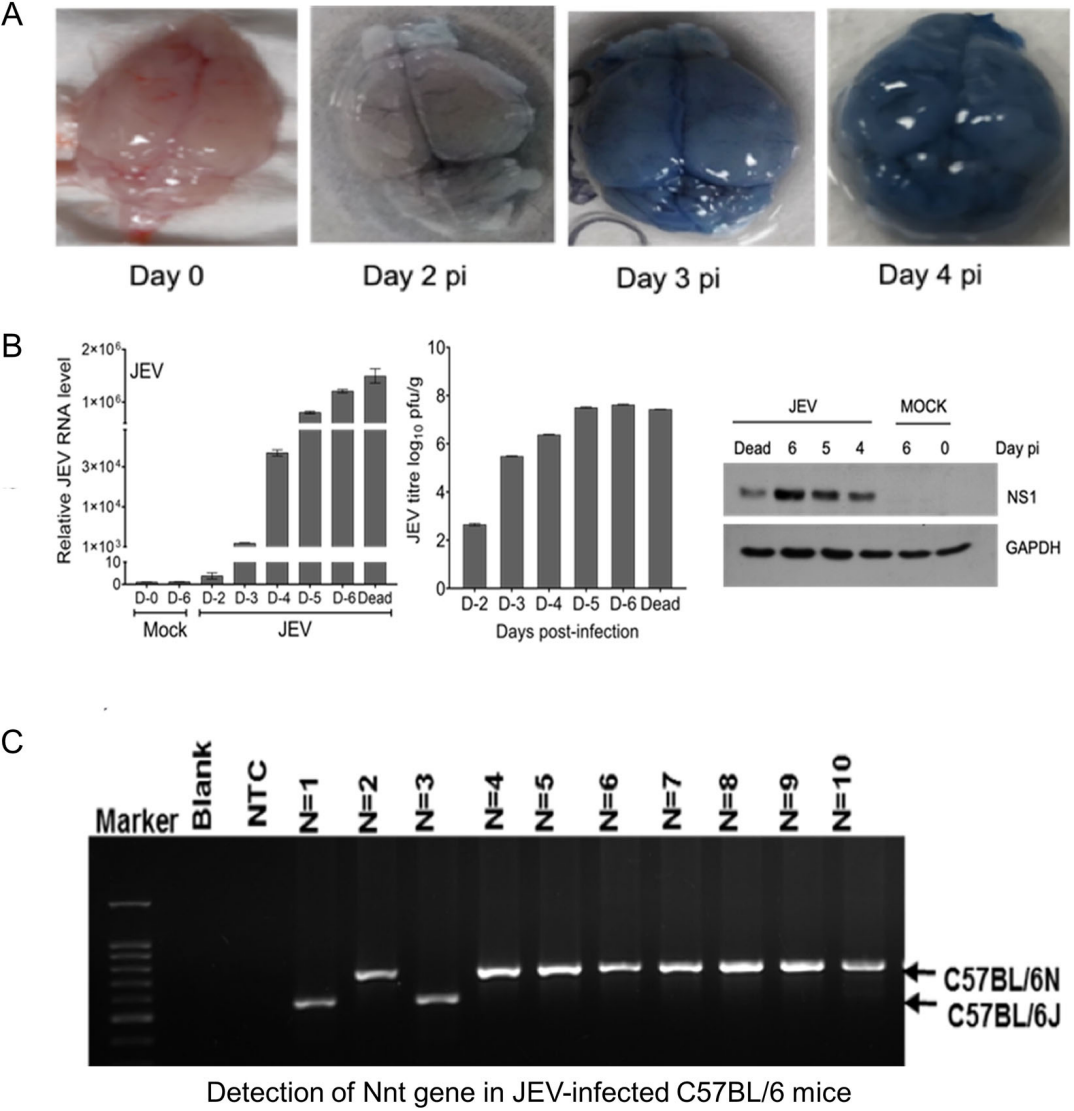
**JEV replication in the mouse brain.** C57BL/6 (3-4 weeks old) mice (*n*=6) were inoculated IP with 100 µl JEV-S3 (10^7^ pfu). (A) Evans Blue dye (200 µl) was injected IP on different days post-infection (pi), and the mouse brain harvested 1 h later. Evans Blue dye-stained brain tissue from JEV-infected mice on different days pi are shown. (B) The level of JEV RNA (left), virus titres (middle) and level of JEV NS1 protein (right) in the brain on different days pi are shown. GAPDH was used as a loading control. (C) Presence of the substrain-specific *Nnt* gene sequence as detected by PCR is shown in C57BL/6 mice infected with JEV.

As shown in Fig. 2A,D, JEV-S3-infected mice exhibited progressive hunchback posture, limbic seizures, limbic weakness and paralysis with unsteady walking (gait), as is usually observed in JEV-infected patients. Because JEV causes encephalitis in humans, and similar neurological symptoms were seen in JEV-S3-infected C57BL/6 mice, we studied the BBB integrity in mice following virus infection. Evans Blue dye was injected IP in C57BL/6 mice 48 h pi, and the mouse brain was harvested 1 h later. Compared to mock-infected control, the brain from the virus-infected mice showed Evans Blue uptake at day 3 pi, suggesting a BBB breach (Fig. 3A). The dye uptake was more intense on subsequent days, meaning further breach of the BBB.

We also studied the kinetics of JEV replication in virus-infected mouse brains (Fig. 3B). Although BBB breaching was not evident on day 2 pi, the infectious virus could be detected at a lesser amount (10^2.6^ pfu/g) in a plaque assay. The virus titres increased gradually until day 6 pi, with peak titres of ∼10^7.6^ pfu/g when mice succumbed to the virus infection. Proper BBB breaching was seen on day 3 onwards. The replication of the virus in the brain was confirmed by western blotting for the JEV non-structural protein NS1, which increased as the virus replication peaked towards day 5-6 pi (Fig. 3B).

Further, we checked whether both the N and J substrains of the C57BL/6 mice were susceptible to JEV-S3 (Fig. 3C). We genotyped the *Nnt* gene of virus-infected mice using substrain-specific primers. The PCR data showed that mice of both the N and J substrains were susceptible to JEV-S3 infection.

Further, to check whether the mouse-adapted JEV could be grown efficiently in the human cells, we infected human hepatoma (Huh7) and human neuroblastoma (SK-N-SH) cells with the mouse-adapted JEV-S3 virus. The JEV-S3-infected cells exhibited viral NS1 expression 24 h post-infection (hpi), suggesting that the human cells were susceptible to the JEV-S3 virus (Fig. S2A,B). We observed a time-dependant increase in the virus titre. Compared to the parent virus (TC), JEV-S3 grew to a significantly higher titre in SK-N-SH cells (Fig. S2C).

### Histopathological evidence of brain inflammation in JEV-S3-infected mouse brain

**Fig. 4.**
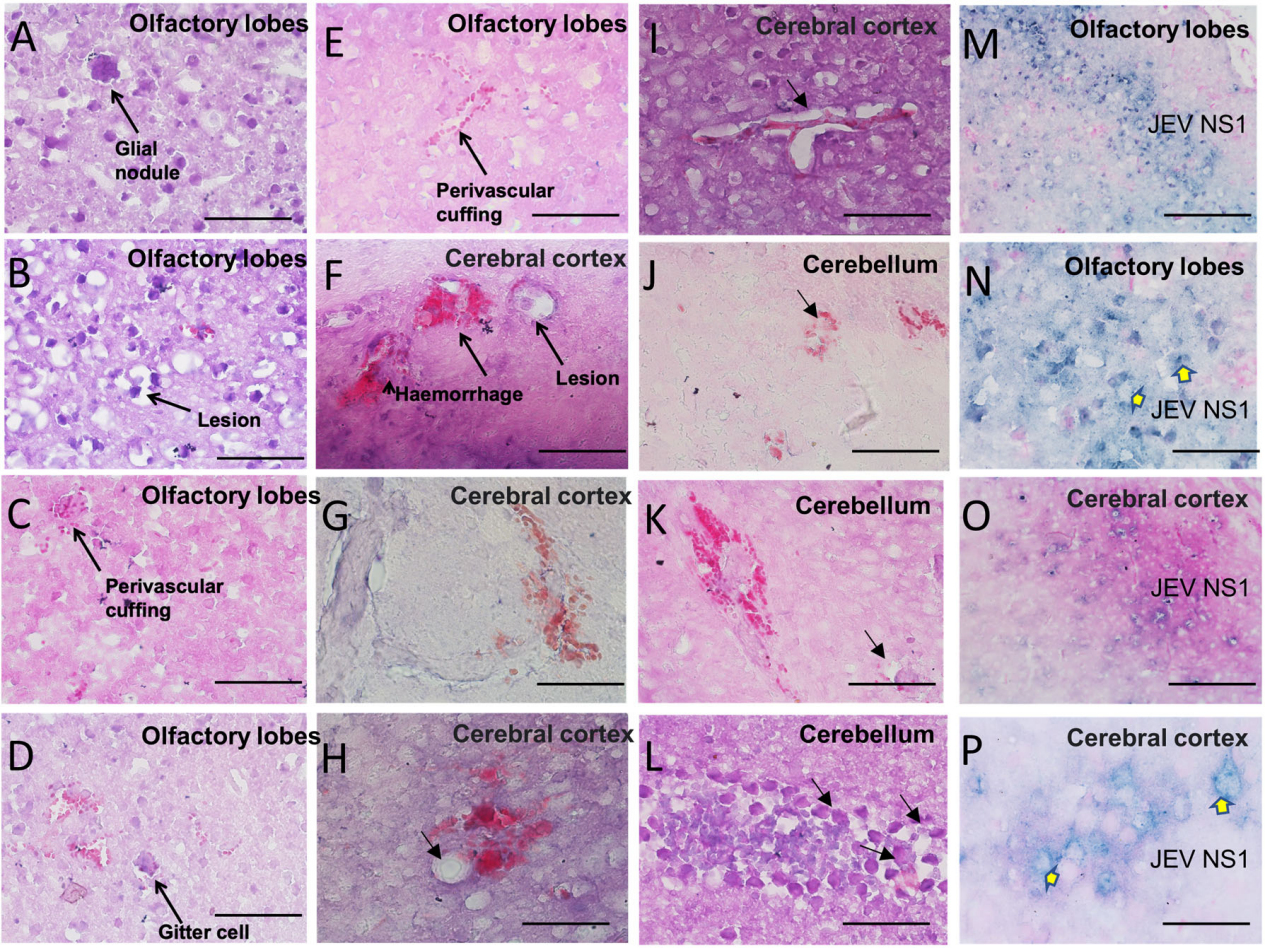
**Pathology of JEV in C57BL/6 mouse model.** Representative Haematoxylin and Eosin-stained slices of brain tissue at 40× magnification (20× magnification in M and O) represent histopathology of brain from C57BL/6 (3-4 weeks old, *n*=3) mice infected with 100 µl JEV-S3 (10^7^ pfu) through the IP route. (A-E) Glial nodule, necrotic lesions and perivascular cuffing are the histologic features demonstrated in the olfactory lobes of JEV-infected brain. (F-L) Perivascular haemorrhage, necrotic lesion and infiltrating cells in the cerebral cortex and cerebellum. Arrow in H indicates a lesion; arrows in L indicate immune cell infiltration. (M-P) Immunoperoxidase staining with Haematoxylin counterstain showed the presence of viral antigens (NS1) in olfactory lobes and cortex. Scale bars: 5 µm.

The Haematoxylin-Eosin blue-stained brain sections of JEV-infected mice were examined under a microscope (Fig. 4). The histopathological studies of different brain regions showed a distinct resemblance of the encephalic lesions observed in humans. Immunohistochemical staining of viral antigen (NS1) was seen in the olfactory lobes (Fig. 4M,N) and cortex (Fig. 4O,P), further confirming infection in the brain. Moreover, glial nodules, ‘punched-out’ necrotic lesions, perivascular cuffing, and the presence of necrotic foci with gitter cells were evident in the olfactory lobes and cerebral cortex (Fig. 4A-E). Apparent haemorrhagic lesion, damage, necrotic foci, mild vascular damage (Fig. 4F-K) and perivenous mononuclear infiltrate were observed in the cortex and cerebellum (Fig. 4I).

**Fig. 5.**
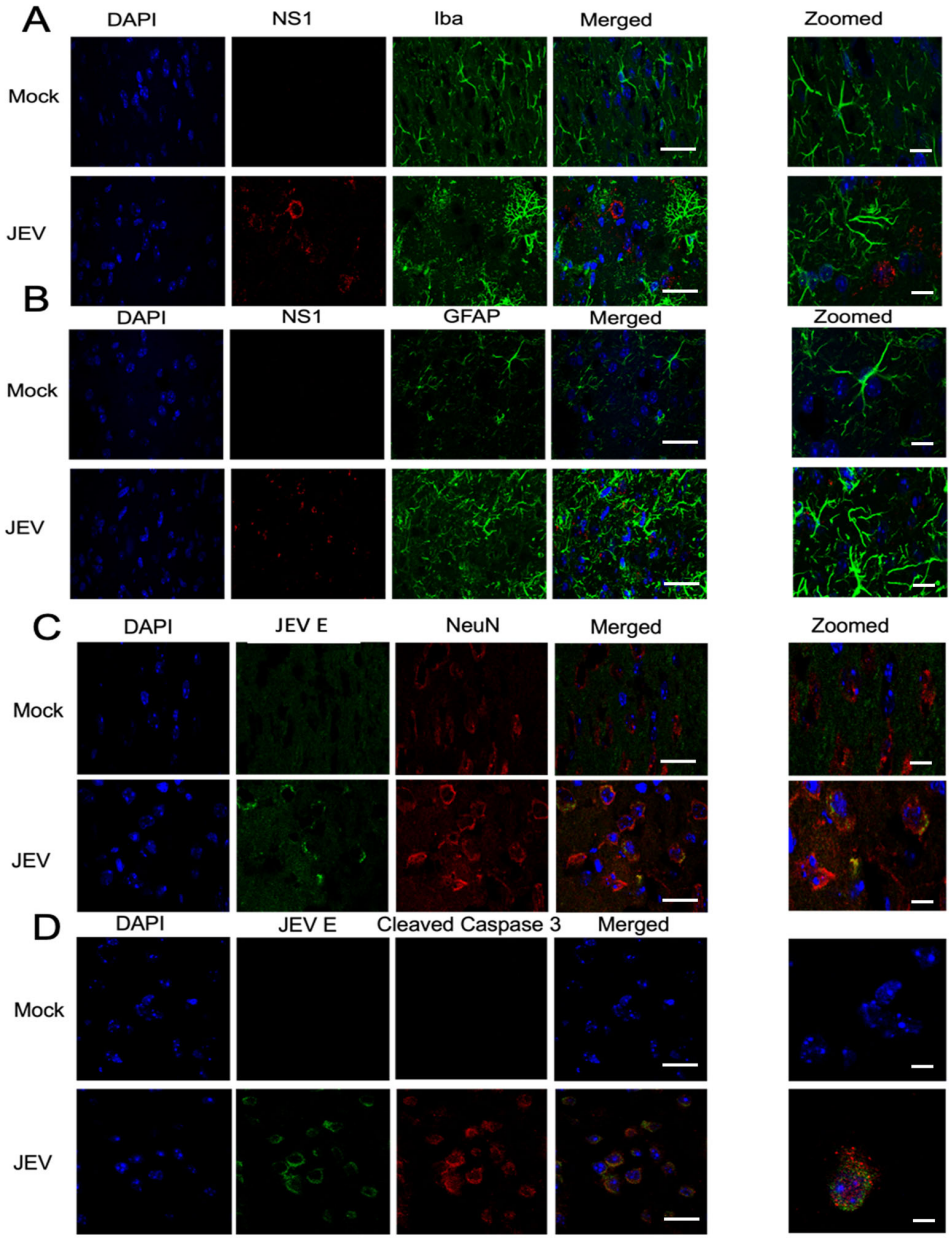
**Microglia, astrocyte and caspase activation in JEV-infected mouse brain.** C57BL/6 (3-4 weeks old) mice (*n*=3) were inoculated IP with 100 µl JEV-S3 (10^7^ pfu), and brain tissues were harvested 24 h post-onset of symptoms. Microglia, astrocytes, neuron and activated caspase-3 were visualized by immunohistochemical staining of the brain sections from mock- and JEV-infected mice using antibodies against IBA1 (A), GFAP (B), NeuN (C) and (cleaved) caspase-3 (D). Viral infection in the brain tissue sections was demonstrated by staining for viral NS1 (A,B) and E (C,D) proteins. Nuclei were stained with DAPI (blue). Scale bars: 10 µm (5 µm in zoomed images).

The astrocytic and microglial responses were assessed by staining the brain sections for GFAP and IBA1. The mock-infected brain sections had relatively shorter length processes, whereas unusual astrocyte staining with long-branched processes was observed in the JEV-infected brain (Fig. 5A). Further immunohistochemical analysis indicated the presence of activated microglia, as evidenced from their long and multiple branches in JEV-infected brain tissue (Fig. 5B). Here, JEV infection of the neuronal cell was evident, with the co-occurrence of JEV Envelope (E) and NeuN protein in the double-stained brain sections (Fig. 5C). In addition, JEV E-positive cells showed activated caspase-3 activation, suggesting possible neuronal damage in the infected brain (Fig. 5D).

**Fig. 6.**
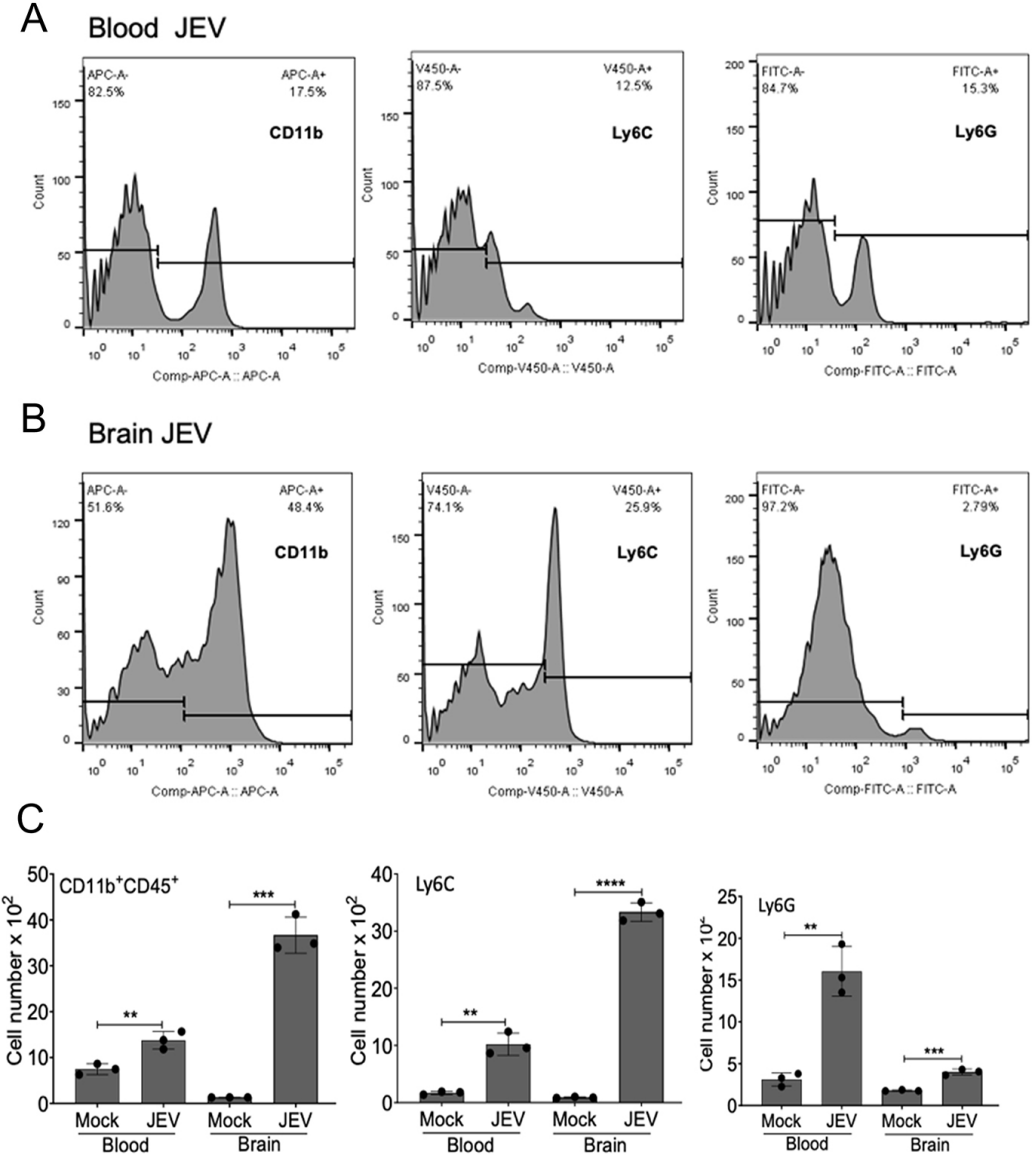
**Flow cytometric analysis of immune infiltrating cells in JEV-S3-infected brain.** C57BL/6 (3-4 weeks old) mice (*n*=3) were inoculated IP with 100 µl JEV-S3 (10^7^ pfu), and brain tissues were harvested 24 h post-onset of symptoms. (A,B) Comparison of cell surface expression of markers CD11b, Ly6C and Ly6G on blood (A) and brain (B) cells from JEV-infected mice. (C) Bar graphs show the number of leukocytes expressing CD45, CD11b (left), Ly6C (middle) and Ly6G (right) in the blood (per 100 µl) and brain tissues per mouse (**P<0.01, ***P<0.001, ****P<0.0001) using unpaired Student's *t*-test. The bars represent mean±s.d. of data from three mice in each group.

Flow cytometry study of mock- and JEV-infected mice demonstrated the immune cell population differences in the blood and brain tissues. CD45 (also known as PTPRC; a haematopoietic cells marker) and CD11b (also known as ITGAM; a pan-myeloid marker) showed an increased presence in the JEV-infected mouse brain compared to the mock. Inflammatory monocyte (Ly6C; also known as LY6C2) and Ly6G (representing granulocytes)-positive cells showed more significant accumulation in the brain and blood in infected mice (Fig. 6A-C).

### JEV infection induces inflammation in the mouse brain

To understand the molecular events that lead to the development of clinical symptoms in JEV-infected C57BL/6 mice, we performed quantitative proteomics of brain tissue from JEV-S3-infected mice in comparison to that from mock-infected mice, employing label-free mass spectrometry (Fig. S1). After normalizing the data against the brain from the mock-infected mice, we observed 58 proteins upregulated significantly (≥2-fold, *P*<0.05) and 60 proteins downregulated significantly (≤0.5-fold, *P*<0.05) in the brain from JEV-infected mice. Pathway enrichment analysis of upregulated genes showed their association with actin cytoskeleton polymerization, interferon-mediated response, antiviral defence response and neuronal death (Fig. S1A). The change in protein level is presented in Fig. S1B. Pathway enrichment analysis showed that the downregulated genes were mostly associated with metabolic processes: metabolism of lipids, amide biosynthetic process, glycosphingolipid metabolism and the glycogen metabolic pathway (Fig. S1B).

**Fig. 7.**
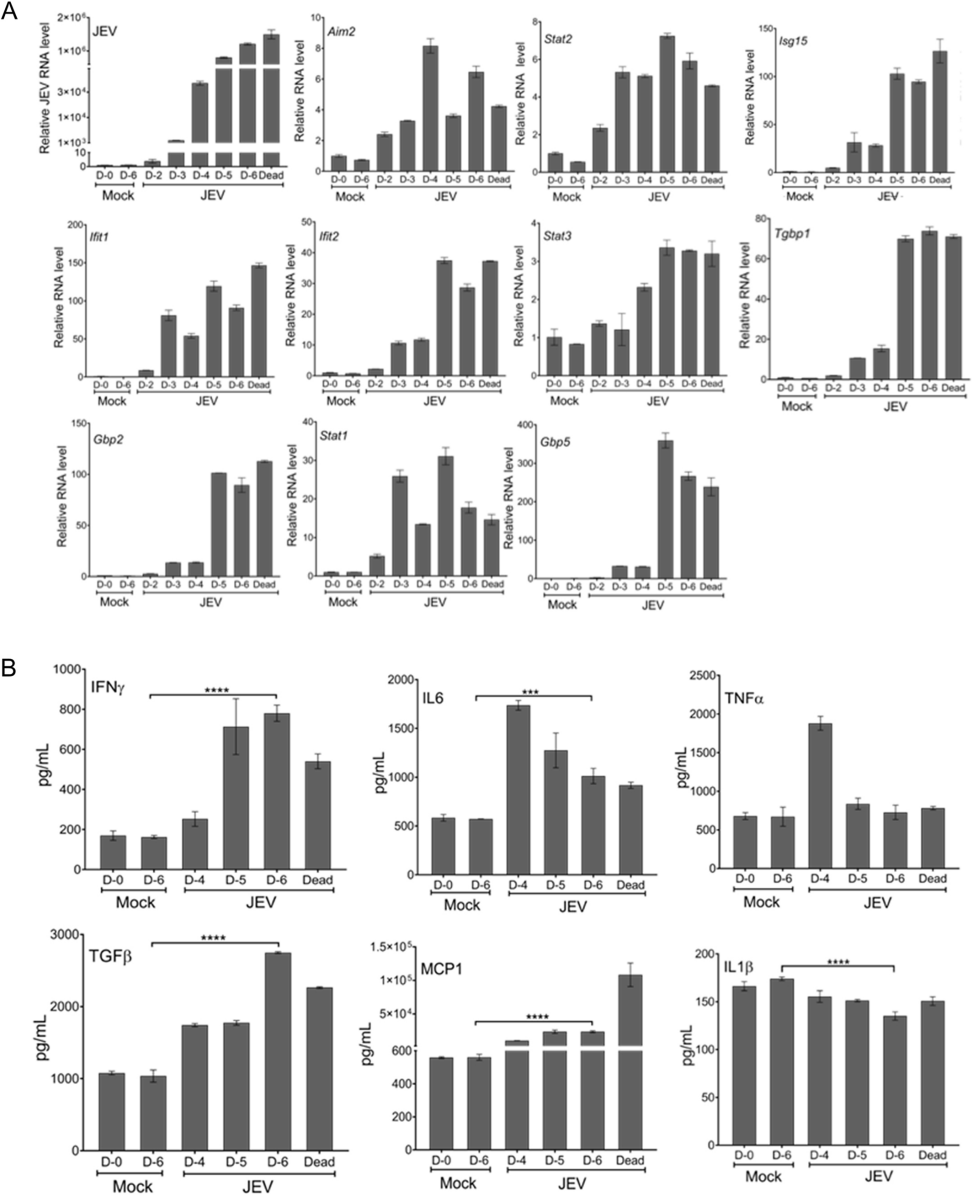
**Expression of interferon-stimulated genes and pro-inflammatory cytokine levels in the JEV-infected mouse brain.** (A) C57BL/6 (3-4 weeks old, *n*=6) mice had inoculated IP with 100 µl JEV-S3 (10^7^ pfu), and brain tissues were harvested from mice at indicated days pi or when mice died. RNA was extracted from the tissue, and qRT-PCR was performed to study gene expression. *Gapdh* mRNA level was used for normalization. (B) C57BL/6 (3-4 weeks old, *n*=6) mice were inoculated IP with 100 µl JEV-S3 (10^7^ pfu), and brain tissues were harvested from mice at indicated days pi or when mice died. Protein lysates were prepared from the tissue, and the levels of cytokines were measured using a cytokine bead array kit. Data were analysed by FCAP and Qognit software. The cytokine levels on day 6 pi were compared between the virus-infected and mock-infected mice using unpaired Student's *t*-test. Values are expressed as mean±s.d. (***P<0.001, ****P<0.0001).

We then studied several genes that were associated with the antiviral response and inflammatory processes by quantitative reverse-transcription polymerase chain reaction (qRT-PCR) in JEV-S3-infected mouse brain harvested at different days pi. The JEV RNA was detectable in the brain as early as day 2 pi, and its level increased gradually as the symptoms developed. There was a significant increase in the expression of interferon-stimulated genes (*Sat1-3*, *Tgbp1*, *Aim2*, *Gbp2*, *Gbp5*, *Isg15*, *Ifit1*, *Ifit2*) (Fig. 7A), as well as upregulation of pro-inflammatory cytokines (IFNγ, TNFα, IL6, TGFβ, IL27), in JEV-infected mice compared to mock-infected mice. Anti-inflammatory cytokines such as IL10 and IL4 were slightly decreased around day 5-6 pi, when the infected mice succumbed to the infection (Fig. 7B).

### JEV genome mutations selected during mouse adaptation

To understand the differences in virulence of the tissue-cultured parent virus and the mouse-passaged JEV-S1 and JEVS3, the virus genome sequences from each of these stages were established. Although no mutations were seen in the 5′- and 3′-non-coding regions and the RNA encoding the capsid and membrane proteins, nucleotide substitutions were seen in the genome sequence encoding the structural protein E and the non-structural proteins NS1, NS2a, NS3, NS4a, NS4b and NS5 (Table 1). Most of these substitutions were in the third position in the codon and did not result in an amino acid change. Two amino substitutions were seen in E protein (K343E and N600K), one substitution in NS3 protein (H2053Y), three substitutions in NS4b protein (R2436K, V2445I, V2453G) and one substitution (V2604I) in NS5 protein (Table 1).

**Table 1.**
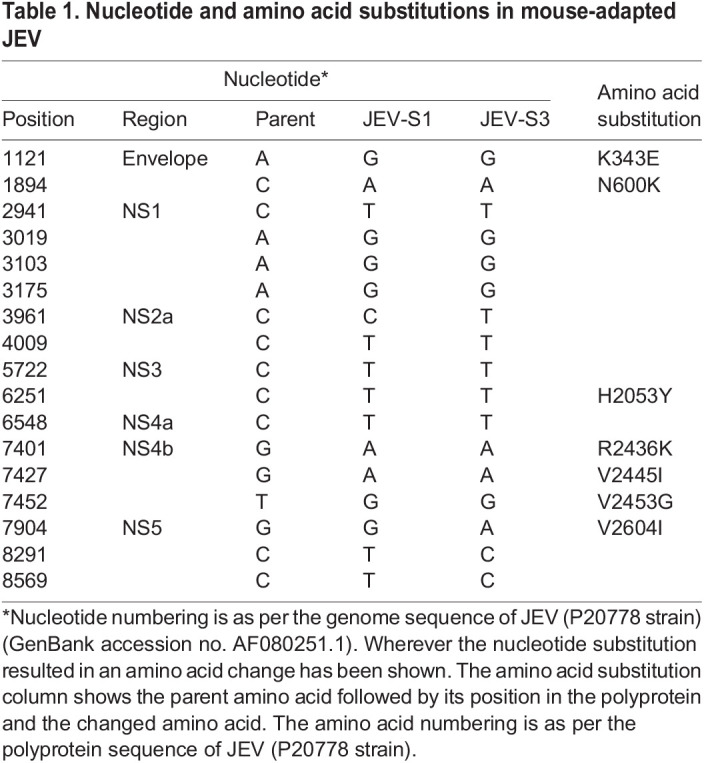
Nucleotide and amino acid substitutions in mouse-adapted JEV

### Use of the mouse model of JEV infection to test antivirals

Ribavirin is known to have broad antiviral activity against RNA viruses (Baz et al., 2017; Kamar et al., 2010; Mejer et al., 2020; Westover et al., 2016). It has been shown to inhibit JEV replication in *in vitro* and *in vivo* studies (Sebastian et al., 2009, 2012) and has been tested as an antiviral to treat JEV-infected patients (Leng et al., 2020; Lv et al., 2018). We validated the utility of the mouse model of JEV infection developed here by testing the antiviral property of Ribavirin. Ribavirin and Ribavirin+JEV-S3 groups were treated with Ribavirin given IP (dose 30 mg/kg) 24 h pi with one dose each day for 7 days. C57BL/6 mice (3-4 weeks old) infected IP with JEV-S3 developed clinical symptoms of JE, and 100% of them succumbed to infection by day 12 pi, with the MST of 11.5 days. However, 30% of JEV-infected mice treated with Ribavirin survived the infection, with the MST of those succumbing to the infection being 14 days, thus demonstrating the antiviral activity of ribavirin and validating the use of the mouse model for antiviral testing against JEV (Fig. S3).

## DISCUSSION

Considering its medical significance, efforts are being made in different laboratories to develop novel antivirals against JEV. In order to test these antiviral agents, appropriate small-animal models are desirable. Previously, a small-animal peripheral challenge model of JEV infection was developed using interferon-deficient AG129 mice. As interferon pathways are defective in the AG129 mice, this model is not ideal for antiviral studies, especially where the antiviral might trigger immune response-related effects (Calvert et al., 2014). By serial passaging in C57BL/6 mice, we have generated a mouse-adapted JEV isolate (JEV-S3) that was used to develop a robust mouse model of JEV infection in 3- to 4-week-old C57BL/6 mice inoculated through the IP route. All mice could be reproducibly infected with JEV-S3. The infected mice developed clinical symptoms, showed upregulation of pro-inflammatory proteins similar to those seen in humans (Ravi et al., 1997) and succumbed to death eventually, with the virus entering the brain and replicating to a high titre. There are multiple substrains of C57BL/6 mice. We checked the susceptibility of B6J and B6N strains to the mouse-adapted JEV and found no obvious differences. Importantly, 3- to 4-week-old BALB/c mice were equally susceptible to infection with JEV-S3 (Fig. 2). The utility of JEV-S3 to test the antiviral efficacy in C576BL/6 mice was demonstrated using Ribavirin, a known inhibitor of JEV replication. The animal model of JEV infection presented here will, thus, be useful for understanding details of pathogenesis and may also be useful for screening the antiviral molecules.

Whereas the 3- to 4-week-old C57BL/6 mice developed clinical symptoms and succumbed to JEV infection with 100% mortality, we observed that older mice (>5 weeks) were less susceptible to lower mortality rates as the age of the mice progressed. This was consistent with previous observations in which older animals (38- to 284-day-old mice) exhibited higher resistance to JEV infection (Miura et al., 1988). Apart from age, the route of inoculation is another key factor for the susceptibility of mice to flaviviruses (Clark et al., 2012). Immunocompetent mice, aged 3-4 weeks, are susceptible to a peripheral challenge with flaviviruses, but, after this time, signs of viremia and infection are not apparent. We observed that passaging of the virus in 3- to 4-week-old mice was required to establish a consistent infection in mice. The blind passaging in the 3- to 4-week-old mice through the IV route probably allowed the virus to circumvent certain host-restricting factors and allowed the adaptation to take place more efficiently, resulting in reproducible infection in adolescent mice even through the IP route.

Most of the JEV animal models have demonstrated the development of clinical symptoms and replication of the virus in the brain and other organs. However, none of the models have been characterized at the molecular level to understand how the virus infection alters the cellular metabolism in the brain, the critical target organ affected during encephalitis. Here, we present, for the first time, the global changes in protein expression level in the JEV-infected mouse brain. It is clear from our proteomics data that the antiviral innate immune pathways were highly active as the mice developed the disease symptoms. Further, as the disease progressed, the antiviral genes were upregulated significantly. These data indicate that our model would be suitable to assess the antiviral drugs, which might have the immunomodulatory mode of action.

In addition to genes of the interferon signalling pathway, actin cytoskeleton polymerization-related genes were also significantly upregulated in the brain of JEV-infected mice. It has previously been shown that actin was required to facilitate the early process of JEV infection in IMR32 cells (Henry Sum, 2015). A novel finding from the present work is the enrichment of neutrophil degranulation pathways during JEV infection of the mouse brain. Previously, matrix metalloproteinase-9 (MMP-9) levels were shown to be related to poor neurological outcomes in humans. MMP-9-positive neutrophil infiltration was shown to be associated with BBB breakdown in humans (Rosell et al., 2008). In fact, the virus was detectable as early as day 2 pi. However, BBB breaching was observed in our mouse model from day 3 onwards. These results are in agreement with the report published by Li et al. (2015), showing the presence of virus in the brain without breach of the BBB during the early time point. Our mouse neuropathology data are similar to those seen in humans (German et al., 2006). As seen in the JEV-infected human brain tissue, we noted increased BBB breaching (as shown by Evans Blue data), and necrotic foci and perivascular cuffing in Haematoxylin-Eosin-stained sections of JEV-infected mouse brain (Fig. 4). We also observed infiltration of leukocytes and granulocytes [as shown by fluorescence-activated cell sorting (FACS) data] in the JEV-infected mouse brain. Immunohistochemical analysis indicated an active astrocytic and microglial response in virus-infected mouse brain, as shown by enhanced GFAP staining and IBA1 expression. Viral protein expression was also evident in the neuronal cells in JEV-infected mouse brain (Fig. 5). Another important observation was that the pro-inflammatory cytokines were highly upregulated as JEV infection progressed towards severity. By contrast, anti-inflammatory cytokine levels were reduced during JEV infection. TNFα, IL6, IL8 and RANTES (also known as CCL5) were identified as important pro-inflammatory cytokines associated with human JEV infections. Winter et al. (2004) correlated an increase in IL6, IL8 and TNFα in cerebrospinal fluid with an increase in RANTES in the plasma of JEV-infected patients with a negative disease outcome. In this study, we saw similar results, with an increase in IL6 in the brain, along with an increase in TNFα.

To understand the genome mutations selected during virus passaging in the mouse, the whole genome of the tissue-cultured parent JEV and the mouse-passaged JEV was sequenced. The JEV-S1 and JEV-S3 genomes showed high levels of sequence identity with the parent virus. For JEV-S3, the identity with the parent virus was 99.73% at the nucleotide level and 99.2% at the amino acid level. A total of 17 nucleotide substitutions were found, seven of which resulted in amino acid substitutions. Repeated passaging of the virus in mice was expected to select mutations, allowing the virus to interact better with the host cell receptor. Thus, mutations in the E protein were expected (Chiou et al., 2012; Leng et al., 2020; Tajima et al., 2010). Indeed, two amino acid substitutions were seen in the E protein. The K343E mutation changed a Lys with a charged basic side chain to Glu with a charged acid side chain. In another mutation, N600K, the amino acid Asn with a polar side chain was replaced by Lys with a charged side chain. These changes could alter the local conformation of E protein, which might have been reflected in the virus adaptation to the mouse receptor. The other amino acid substitutions in the non-structural proteins could affect virus replication. It may be of interest to note that the maximum mutations were selected in the NS4b protein. Considering that this is a small protein, three amino acid substitutions in NS4b during its mouse adaptation indicate its important role in virus pathogenesis. Mutations in the NS4 protein of the dengue virus, another flavivirus, were reported during its adaptation to mice (Grant et al., 2011; Zmurko et al., 2015). These mutations may have an important role in viral fitness. The role of the mutations in virus pathobiology, acquired by JEV during mouse adaptation, needs further study.

In summary, we present a robust mouse model of JEV infection in which 3- to 4-week-old C57BL/6 mice reproducibly showed clinical symptoms when injected IP with the mouse-adapted JEV. We present, for the first time, an in-depth characterization of the model, detailing the proteins dysregulated in JEV-infected mouse brain, the critical organ involved in encephalitis during the viral disease progression. The robust and reproducible mouse model and the information on the brain proteome during JEV infection will be useful in designing novel antivirals. This JEV-S3 strain can infect both C57BL/6 and BALB/c mice efficiently, thus providing a better choice for the scientific community to use this strain for a broader-range knockout model using BALB/c or C57BL/6 mice.

## MATERIALS AND METHODS

### Ethics and biosafety statements

Animal experiments were approved by the Translational Health Science and Technology Institute (THSTI) Animal Ethics Committee (approval no. IAEC/THSTI/2018-2). Animals were handled in strict accordance with the guidelines of the Committee for Control and Supervision of Experiments on Animals, Ministry of Environment and Forestry, Government of India. They were kept under a 12 h light/dark cycle with constant temperature and humidity; food and water supply were provided *ad libitum*. All the *in vitro* and animal experiments were performed strictly under BSL-2 containment as per the ‘Regulations and Guidelines for Recombinant DNA Research and Biocontainment, 2017’, notified by the Department of Biotechnology, Government of India, duly approved by the Institutional Biosafety Committee (approval no. IBSC/01018/2017 #66/17).

### Generation and purification of cell culture-grown JEV

JEV P20778 strain (GenBank accession no. AF080251) was grown in C6/36 cells to a titre of 2.25×10^8^ pfu/ml. Briefly, the C6/36 cell line (CRL-1660, American Type Culture Collection, Manassas, VA, USA) was grown in L-15 medium (Invitrogen, Carlsbad, CA, USA) containing 10% foetal bovine serum (FBS; Invitrogen) supplemented with 1% penicillin-streptomycin and 2 mM glutamine (PSG; Invitrogen) at 28°C without CO_2_. To amplify the virus, C6/36 cells were seeded at 40% confluency in a T175 flask. The next day, the cell monolayer was washed with PBS, and JEV inoculum at 0.01 multiplicity of infection (MOI) was added with intermittent shaking for 1 h at 28°C. The virus inoculum was removed, and the cells were washed twice with PBS followed by incubation under L-15 medium supplemented with 2% FBS, 1% penicillin-streptomycin and PSG for 72 h at 28°C. The virus in the culture supernatant was collected and purified using the sucrose gradient ultra-centrifugation method. Briefly, the culture supernatant from virus-infected cells was centrifuged at 2000 ***g*** for 10 min to remove the cell debris. NaCl was added to a concentration of 0.3 M to the supernatant. PEG-6000 (Sigma-Aldrich, St Louis, MO, USA) was then mixed to a concentration of 8% (w/v) and left for overnight incubation with gentle stirring at 4°C. The virus was pelleted by centrifugation at 15,000 ***g*** for 30 min at 4°C, and suspended in TES buffer (0.01 M Tris-HCl, 0.002 M EDTA, 0.15 M NaCl; pH 7.2) that was 1/100th of the virus stock volume. The suspension was centrifuged at 13,000 ***g*** for 4 min at room temperature. The supernatant containing the virus was loaded onto a 20% sucrose cushion and centrifuged at 13,000 ***g*** for 3 h at 4°C. The pellet was resuspended in PBS (1/100th of virus stock volume), aliquoted and stored at −80°C.

### Mouse adaptation of JEV

C57BL/6 mice pups (3-4 days old) of either sex were injected IC with 10^3^ pfu of JEV P20778 and housed with their mother. The pups developed paralysis symptoms at 72-96 hpi. At this time, brain tissue was collected to prepare a 40% suspension in minimal Eagle medium (MEM; Invitrogen) followed by centrifugation at 380 ***g*** for 10 min at 4°C. The supernatant was passed through a 0.22 µm sterile filter. Titration of the virus in the brain suspension was done in porcine kidney (PS) cells by plaque assay. In brief, PS cells obtained from the National Centre for Cell Science, Pune, India, were maintained at 37°C with 5% CO_2_ in MEM with 10% FBS, 1% PSG. PS cells were seeded at 60% confluency per well. Cells were washed with 1× PBS followed by incubation with 10-fold serially diluted virus stock prepared in serum-free MEM for 1 h, incubated at 37°C with gentle rocking. Following this, the cells were washed with 1× PBS and overlaid with 2 ml agarose type VII (Sigma-Aldrich) in 2× MEM (1:1 ratio) with 10% FBS. After 4 days, cells were fixed with 3.7% formaldehyde (Sigma-Aldrich). The overlay plugs were removed, and cells were stained with 1% Crystal Violet (Sigma-Aldrich) for 5 min. Plaques were counted, and virus titre was calculated in pfu/ml by the following formula: virus titre (pfu/ml)=average count of pfu/volume of infection (ml)×dilution factor.

### Virus passaging in mice

The C6/36 cell-grown JEV P20778 (parent virus) (Vrati, 2000) was serially passaged four times in the pups to obtain the JEV-S1. C57BL/6 mice (3-4 weeks old) of either sex were injected IV with 10^8^ pfu of JEV-S1 through tail vein injection. Following the onset of symptoms, brain tissue was collected for virus preparation and titrated as above. The virus was serially passaged three times in 3- to 4-week-old mice to obtain the S2 JEV (JEV-S2). C57BL/6 mice pups (3-4 days old) of either sex were then injected IC with 10^3^ pfu of JEV-S2. The pups developed paralysis symptoms at 72-96 hpi. At this time, the brain was collected to prepare the virus and titrated as above. This virus was designated as JEV-S3.

### JE clinical sign scoring

Mice injected with JEV were monitored twice daily for body weight and the following signs. The scores given for the different clinical symptoms were as follows: 1, piloerection; 2, body stiffening, piloerection; 3, body stiffening, piloerection, restriction of movement; 4, body stiffening, piloerection, restriction of movement, hind limb paralysis; 5, body stiffening, piloerection, restriction of movement, hind limb paralysis, whole-body tremor.

### BBB disruption study

Evans Blue extravasation assay was performed to study the change in BBB permeability. C57BL/6 mice were injected IP with 0.2 ml 1% Evans Blue dye (Sigma-Aldrich) prepared in sterile PBS. After 1 h of dye circulation, mice were sacrificed by cervical dislocation to remove the brain and image captured.

### Brain histology and immunohistochemistry

Mice were anaesthetized with ketamine/xylazine and transcardially perfused with cold PBS followed by 4% paraformaldehyde. Brains were extracted and stored in 4% paraformaldehyde (Sigma-Aldrich) for 24 h for fixation and then left in 30% sucrose until immersion. Brains were then washed with PBS, embedded in OCT (Tissue Tek, St Louis, MO, USA) and stored at −20°C until further use. Sections (12 μm) of the brain were made with the help of a cryostat (Microm HM550, Thermo Fisher Scientific, Runcorn, UK) and mounted on slides. For Haematoxylin-Eosin staining, sections were washed twice with PBS and stained with Haematoxylin (Sigma-Aldrich) followed by incubation in 0.5% HCl. Brain sections were washed and treated with a gradient of alcohol (30%, 50% and 70%) before staining with Eosin (Sigma-Aldrich). The sections were then treated with a gradient of alcohol (90% and 100%), rinsed with xylene and mounted with DPX (Sigma-Aldrich). For focus-forming assay, after permeabilization and blocking, brain sections were incubated with primary antibody against JEV E glycoprotein (1:20; ab41671, Abcam, Cambridge, MA, USA) for 2 h and horseradish peroxidase-conjugated secondary antibody for 1 h followed by incubation for 20 min with TrueBlue™ Peroxidase Substrate (SERACARE, Milford, MA, USA). Brain sections were counterstained with Eosin and mounted with DPX (Sigma-Aldrich), then examined with an Eclipse Ti (Nikon, Melville, NY, USA) imaging system. Images were captured at 20× and 40× magnification and analysed using NIS-Element AR 4.6 software.

For immunohistochemistry, the antigen retrieval process was performed at 95°C using antigen unmasking solution (Vector Laboratories, Burlingame, CA, USA). After permeabilization with 0.1% Triton X-100 and blocking with 10% bovine serum, the sections were incubated overnight with a primary antibody against antigens. The primary antibodies used were as follows: in-house rabbit polyclonal antibody against JEV NS1 protein (1:1000), mouse anti-JEV E glycoprotein (1:20; ab41671, Abcam), mouse anti-IBA1 antibody (1:100; MABN92, EMD Millipore, Darmstadt, Germany), mouse anti-GFAP (1:50; MAB3402, EMD Millipore), rabbit anti-NeuN (1:100; ABN78, EMD Millipore) and rabbit anti-cleaved caspase-3 (1:50; 9661S, Cell Signaling Technology, Beverly, MA, USA). After washing with PBS, the sections were incubated for 1 h with fluorochrome-conjugated secondary antibodies: Alexa Fluor 568 goat anti-rabbit (1:500; A11011, Invitrogen), Alexa Fluor 488, goat anti-mouse (1:500; A11029, Invitrogen), Alexa Fluor 568 goat anti-mouse (1:500; A11004, Invitrogen) and Alexa Fluor 488, goat anti-rabbit (1:500; A11008, Invitrogen). Following a 1 h wash with PBS, 4′,6-diamidine-2′-pheynylindole dihydrochloride (DAPI; Invitrogen) was used to stain the nuclear DNA. Images were acquired at 60× magnification using a confocal microscope (Olympus, FV 1000) and analysed with FluoView (FV 31S-SW) software.

### Blood immune cell isolation and flow cytometry analysis

Blood was collected from mice through the retro-orbital route and added to ACK lysis buffer (BioLegend, San Diego, CA, USA) to remove erythrocytes as per the manufacturer's protocol. Isolated lymphocytes were incubated with TruStain FcX™ (anti-mouse CD16/32) antibody (BioLegend) for 15 min at 4°C to block the Fc receptors and then stained with fluorochrome-conjugated antibodies (1:100; APC-CD11b, FITC-CD45, Miltenyi Biotec, Gladbach, Germany), V450-Ly6C (1:50; Invitrogen, Vilnius, Lithuania), FITC-Ly6G (1:50; BioLegend). Cells were then rinsed with FACS buffer, run on BD FACS Verse (BD Biosciences, San Jose, CA, USA) and analysed using BD FACS Suitev1.0.6 (BD Biosciences) and FlowJoV10 (FlowJo LLC) software.

### Immune cell isolation from brain and flow cytometry analysis

Mice were anaesthetized with ketamine and xylazine and perfused with ice-cold PBS. Brains were removed and homogenized with a Dounce homogenizer in cold HBSS buffer (Sigma-Aldrich). Brain homogenate was resuspended to prepare 30% isotonic Percoll (Sigma-Aldrich), which was over-layered on 70% isotonic Percoll. The gradient was then centrifuged at 500 ***g*** for 25 min at 18°C. Mononuclear cells were collected from the 30/70% interface and washed with PBS. To identify microglia, monocytes and granulocytes, isolated mononuclear cells were first pre-incubated with mouse anti-CD16/32 antibody (1:50; TruStain FcX™, BioLegend) for 15 min at 4°C to block Fc receptors, and then stained with fluorochrome-conjugated antibodies (1:100; APC-CD11b, FITC-CD45, Miltenyi Biotec), FITC-Ly6C (1:50; Invitrogen, Vilnius, Lithuania), V450-Ly6G (1:50; BioLegend). Cells were then rinsed with FACS buffer, run on BD FACS Verse) and analysed using BD FACS Suitev1.0.6 and FlowJoV10 software.

### RNA extraction and qRT-PCR

Total RNA was extracted from the tissue samples using an RNeasy Mini kit (Qiagen, Hilden, Germany) following the manufacturer's protocols. cDNA was prepared in two steps using a GoScript™ Reverse Transcription System (Promega, Madison, WI, USA). Gene expression was quantified by qRT-PCR assay in triplicate using SYBR green DNA-binding fluorescent dye (SYBR premix Ex Taq, Takara, Otsu, Shiga, Japan) on a Quanta Studio 6 Flex Real-Time PCR System (Applied Biosystems, Foster City, CA, USA). Relative expression of a gene in JEV-infected cells was calculated using the Ct method with mock-infected cells as the reference and *Gapdh* as an internal control.

### Immunoblotting

Mouse brain tissue was homogenized in Tris-HCl buffer (pH 7.5) with 1 mM phenylmethylsulfonylfluoride (Sigma-Aldrich) and protein lysate prepared. The protein concentration was quantified by the bicinchoninic acid assay (Pierce, Rockford, IL, USA). Equal amounts of protein (30 μg) were separated by 10% SDS-PAGE and transferred to a nitrocellulose membrane. The membrane was probed with either rabbit anti-GAPDH antibody (1:10,000; GTX100118, GeneTex, Irvine, CA, USA) or in-house rabbit polyclonal antibody against JEV NS1 protein (1:10,000). For chemiluminescence detection, luminol reagent (Santa Cruz Biotechnology, Dallas, TX, USA) was used, and the signal was visualized in a ChemiDoc™ XRS System (Bio-Rad, Hercules, CA, USA). Densitometric analysis of the blot was performed using ImageJ software to determine the signal intensity.

### Cytokine bead array (CBA)

CBA was performed to quantitatively measure the cytokine levels for IL6, TNFα, IFNγ, MCP1, IL12p70, IL10 using a mouse inflammation kit (BD Biosciences) and TGFβ, IL27, IL23, IL1β (BioLegend) in brain lysate, according to the manufacturers’ protocols, and analysed using CBA analysis software (FCAP3.0.1 and Qognit). The number of cytokines detected in the lysates was measured against the standard curve obtained from the defined concentration of protein.

### Sample processing for proteomic analysis

The brain lysate prepared as above (100 µg) was reduced with 1 M DTT (Sigma-Aldrich) and alkylated with 1 M iodoacetic acid (Sigma-Aldrich) for 30 min each at room temperature followed by acetone precipitation for 1 h at −80°C. Samples were centrifuged at 13,684 ***g*** for 15 min to pellet down the precipitate, which was washed with chilled acetone. Ammonium bicarbonate (100 mM, Sigma-Aldrich) with trypsin (V511A, Promega; enzyme to protein ratio 1:100) was added to resuspend the precipitate, mixed at 34 ***g*** for 1 min, then incubated at 37°C for 18 h. The digested peptides were cleaned up by OASIS SPE cartridge at 14,000 ***g*** for 10 min. Peptides were eluted with ammonium bicarbonate (100 mM), acidified with 0.1% formic acid and concentrated to 10 μl by speed vac. Liquid chromatography with tandem mass spectrometry was done using an AB SCIEX Triple TOF 5600. A spectral data-dependent acquisition library was generated for peptide identification, and identified proteins were quantitated using SWATH AB SCIEX software. Proteins were selected for further study based on a 5% false-discovery rate cut-off and a minimum of two peptides per protein.

### Bioinformatics analysis

The freely available software package Metascape was used for the bioinformatics analysis of the mass spectrometric findings generated in this study. Proteins with altered expression in JEV-infected brain samples were grouped based on their protein class using the database of protein families.

### JEV genome sequencing

The JEV RNA was isolated using the RNA isolation kit (Qiagen) and used as a template for cDNA synthesis using SuperScript™ IV (Invitrogen, Vilnius, Lithuania) according to the manufacturers’ instructions. For PCR amplification of viral genomic cDNA, a Taq DNA Polymerase High-Fidelity kit (Invitrogen, Vilnius, Lithuania) was used. The PCR product was used for Sanger sequencing on a 3500 Genetic Analyzer (Thermo Fisher Scientific). Data were analysed using BioEdit software. Primer sequences used for sequencing were as follows:

JWF, 5′-GCTCGAGAAGTTTATCTGTGTGAACT-3′; JE2R, 5′-GCCAAAGCAATTGATCGGTCTCGT-3′; JE4F, 5′-TATGAGCCATCGCCAAGCTCAACT-3′; JE5F, 5′-AGAACCAGGGAAGGCTGCAGTAAA-3′; JE6R, 5′-CGTTCTTGATGAGAGTCCAGGCAA-3′; JE7F, 5′-CGTTCCTCGTCAACCCTAATGTCACT-3′; JE8R, 5′-CATGACCTTGACCACTTTGTGCCT-3′; JE3F, 5′-TGGTGGTGCCTTCAGAACACTCTT-3′; JE3R, 5′-ACAGCAATCAAAGCAAGGTGCAGG-3′; JE9F, 5′-CGTGACATAGCAGGAAAGCAAGGA-3′; JWR1, 5′-AGATCCTGTGTTCTTCCTCACCACCA-3′.

### Genotyping for C57BL/6 J/N substrain discrimination

To genotype C57BL/6 for substrain detection, the tails of JEV-infected mice were snipped (2-3 mm) and incubated in tail buffer (5 M NaCl, 1 M Tris-HCl pH 8, 0.5 M EDTA, 20% SDS and 1 mg/ml proteinase K) overnight at 56°C. Genomic DNA was purified and suspended in 1× TE buffer (1 M Tris-HCl, 0.5 M EDTA, pH 8). DNA was quantified using NanoDrop™ 2000/2000c Spectrophotometers (Thermo Fisher Scientific, Waltham, MA, USA) followed by PCR using a DyNAzyme EXT™ PCR Kit (Thermo Fisher Scientific) according to the manufacturer's protocol. Agarose gel (1.5%; Sigma-Aldrich) with EtBr (0.2-0.5 µg/ml; Thermo Fisher Scientific) was run in 1× TAE buffer pH 8.3 (40 mM Tris, 20 mM acetic acid, 1 mM EDTA) to resolve the PCR amplicon at 100 V. Gel images were captured using Gel Doc and analysed using ImageJ software. The primers used for mouse substrain identification using the nicotinamide nucleotide transhydrogenase (*Nnt*) gene were a kind gift from Dr Praful Taylor (National Institute of Immunology, New Delhi, India). The nucleotide sequences of the primers used were as follows: common primer 5′-GTAGGGCCAACTGTTTCTGCATGA-3′; wild-type primer (J substrain-specific), 5′-GGGCATAGGAAGCAAATACCAAGTTG-3′; mutant-type primer (N substrain-specific), 5′-GTGGAATTCCGCTGAGAGAACTCTT-3′.

### Statistical analysis

Statistical analysis was performed using Prism version 8.3.1 (GraphPad Software, San Diego, CA, USA). We determined significance for flow cytometric analysis of immune cell count and cytokine levels of day 6 JEV-infected mice and control groups using an unpaired Student's *t*-test. Values are expressed as the mean±s.d., with a significance level of *P*<0.05. MST was analysed by plotting the Kaplan–Meier curve using a log-rank test.

## Supplementary Material

Supplementary information
