# Supplementary material for: Development and characterization of an animal model of Japanese encephalitis virus infection in adolescent C57BL/6 mouse
Source: Dis Model Mech. 2021 Oct 22;14(10):dmm049176. doi: 10.1242/dmm.049176 (PMC8543065; doi:10.1242/dmm.049176)
Supplement: Supplementary information [file dmm-14-049176-s1.pdf]

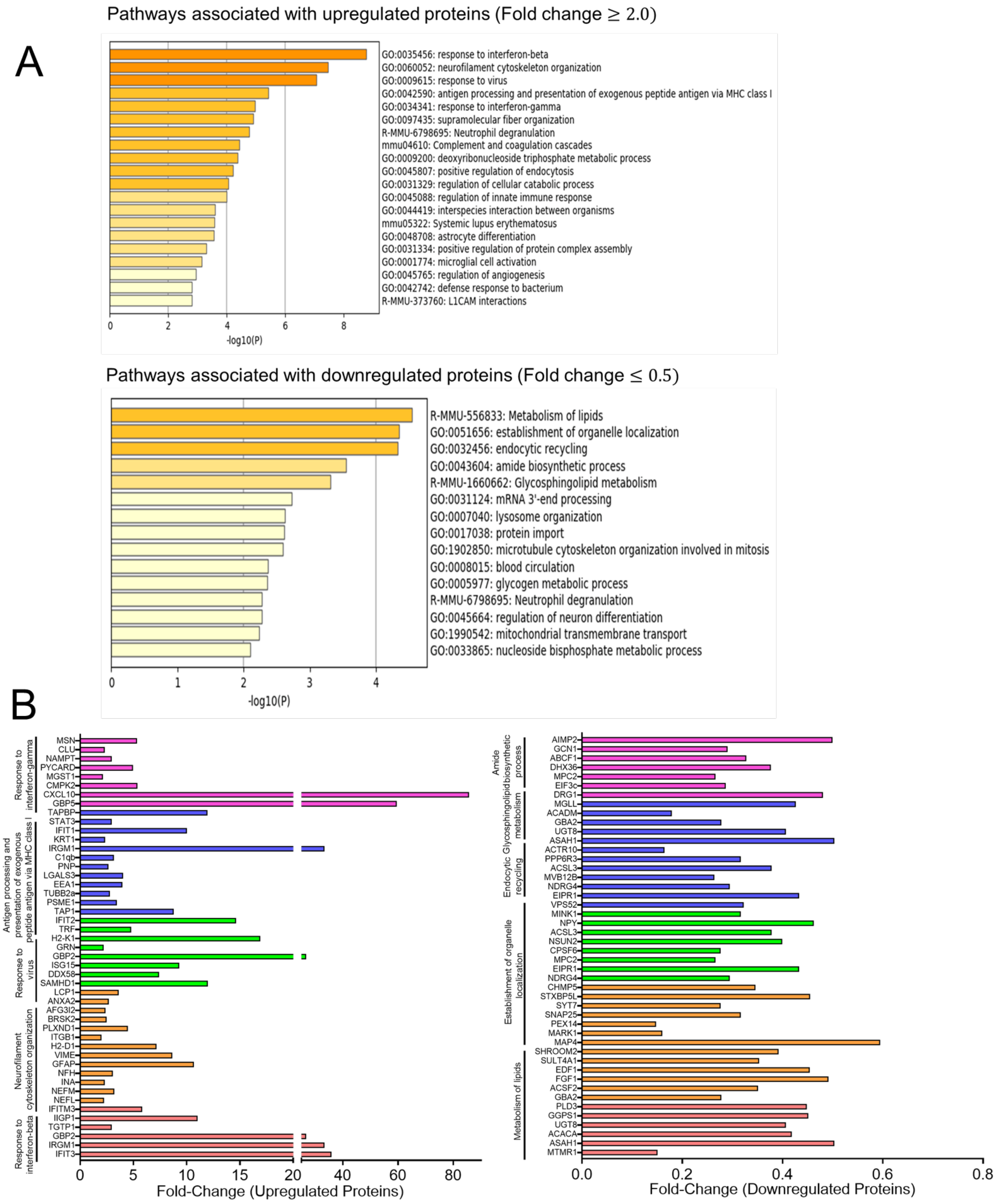
Pathways associated with upregulated proteins (Fold change ≥ 2.0)
A
GO:0035456: response to interferon-beta
GO:0060052: neurofilament cytoskeleton organization
GO:0009615: response to virus
GO:0042590: antigen processing and presentation of exogenous peptide antigen via MHC class I
GO:0034341: response to interferon-gamma
GO:0097435: supramolecular fiber organization
R-MMU-6798695: Neutrophil degranulation
mmu04610: Complement and coagulation cascades
GO:0009200: deoxyribonucleoside triphosphate metabolic process
GO:0045807: positive regulation of endocytosis
GO:0031329: regulation of cellular catabolic process
GO:0045088: regulation of innate immune response
GO:0044419: interspecies interaction between organisms
mmu05322: Systemic lupus erythematosus
GO:0048708: astrocyte differentiation
GO:0031334: positive regulation of protein complex assembly
GO:0001774: microglial cell activation
GO:0045765: regulation of angiogenesis
GO:0042742: defense response to bacterium
R-MMU-373760: L1CAM interactions
0
2
4
6
8
-log10(P)
Pathways associated with downregulated proteins (Fold change ≤ 0.5)
R-MMU-556833: Metabolism of lipids
GO:0051656: establishment of organelle localization
GO:0032456: endocytic recycling
GO:0043604: amide biosynthetic process
R-MMU-1660662: Glycosphingolipid metabolism
GO:0031124: mRNA 3'-end processing
GO:0007040: lysosome organization
GO:0017038: protein import
GO:1902850: microtubule cytoskeleton organization involved in mitosis
GO:0008015: blood circulation
GO:0005977: glycogen metabolic process
R-MMU-6798695: Neutrophil degranulation
GO:0045664: regulation of neuron differentiation
GO:1990542: mitochondrial transmembrane transport
GO:0033865: nucleoside bisphosphate metabolic process
0
1
2
3
4
-log10(P)
B
Response to interferon-gamma
MSN
CLU
NAMPT
PYCARD
MGST1
CMPK2
CXCL10
GBP5
Antigen processing and presentation of exogenous peptide antigen via MHC class I
TAPBP
STAT3
IFIT1
KRT1
IRGM1
C1qb
PNP
LGALS3
EEA1
TUBB2a
PSME1
TAP1
IFIT2
TRF
H2-K1
Response to virus
GRN
GBP2
ISG15
DDX58
SAMHD1
LCP1
Neurofilament cytoskeleton organization
ANXA2
AFG3l2
BRSK2
PLXND1
ITGB1
H2-D1
VIME
GFAP
NFH
INA
NEFM
NEFL
IFITM3
Response to interferon-beta
IIGP1
TGTP1
GBP2
IRGM1
IFIT3
0
5
10
15
20
40
60
80
Fold-Change (Upregulated Proteins)
Amide biosynthetic process
AIMP2
GCN1
ABCF1
DHX36
MPC2
EIF3c
Glycosphingolipid metabolism
DRG1
MGLL
ACADM
GBA2
UGT8
ASAH1
Endocytic recycling
ACTR10
PPP6R3
ACSL3
MVB12B
NDRG4
EIPR1
Establishment of organelle localization
VPS52
MINK1
NPY
ACSL3
NSUN2
CPSF6
MPC2
EIPR1
NDRG4
CHMP5
STXBP5L
SYT7
SNAP25
PEX14
MARK1
MAP4
Metabolism of lipids
SHROOM2
SULT4A1
EDF1
FGF1
ACSF2
GBA2
PLD3
GGPS1
UGT8
ACACA
ASAH1
MTMR1
0.0
0.2
0.4
0.6
0.8
Fold-Change (Downregulated Proteins)

Fig. S1. Proteins dysregulated in JEV-infected mouse brain. C57BL/6 mice (3-4 weeks, n=3) were mock-infected or injected IP with 100 µl JEV-S3 (10^7 pfu). Mice were sacrificed and brain tissue harvested on day six when clinical symptoms became apparent. Protein lysates of the brain tissue were prepared to perform quantitative proteome analysis. Pathway enrichment of proteome profile was carried out by Metascape software. (A) Pathways associated with upregulated and downregulated proteins are shown. (B) The bar plots show fold-change in the expression of different proteins associated with upregulated (fold-change ≥2) and downregulated (fold-change ≤0.5) pathways. IP= Intraperitoneal

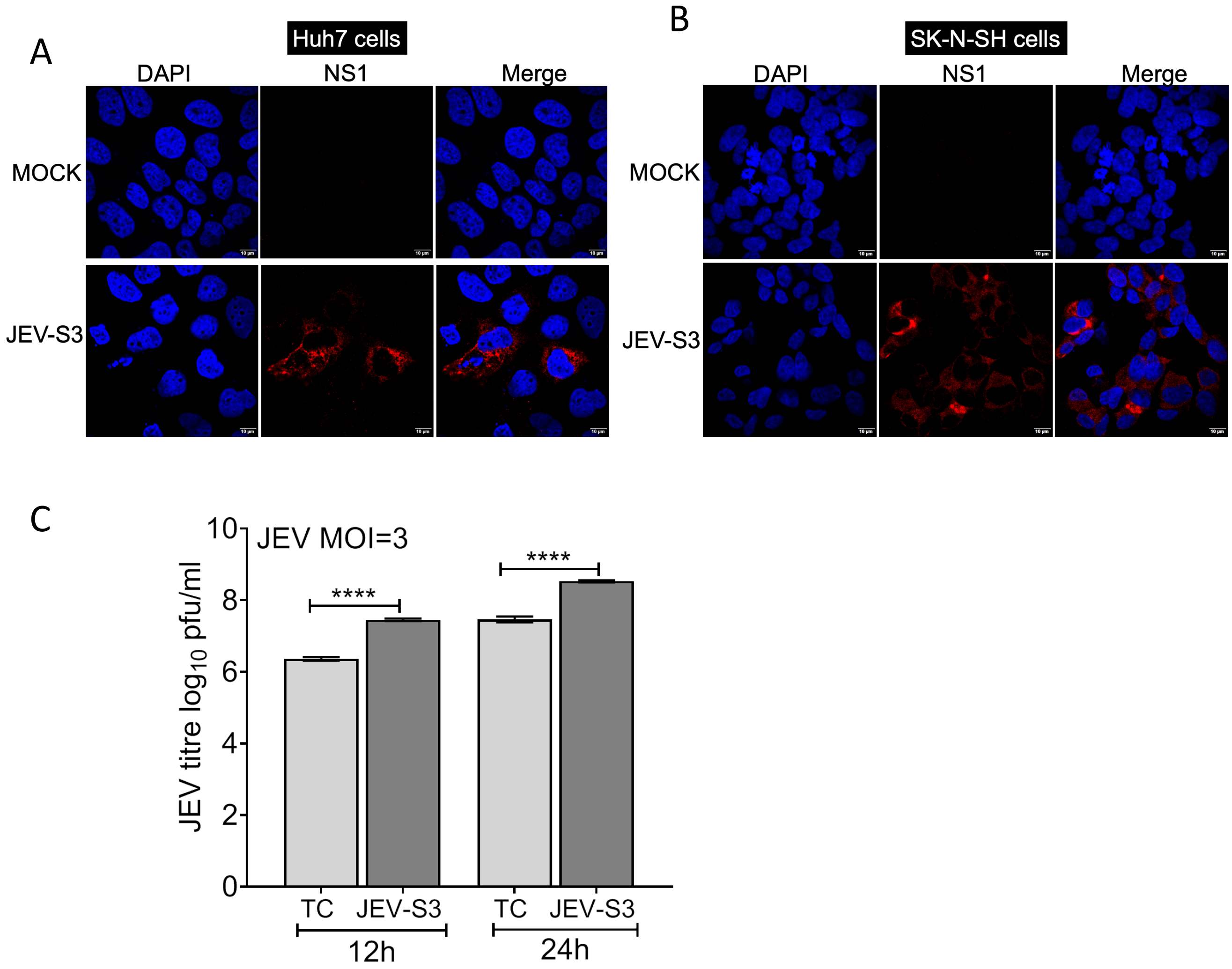

Fig. S2. Infection of human cells with JEV-S3 virus. (A) Huh7 and SK-N-SH cells infected with JEV-S3 at MOI=3, were stained for JEV antigen (NS1, red) and DAPI (Nucleus, blue) 24h post-infection. (B) The SK-N-SH cells were infected with either Tissue culture (TC) grown virus or JEV-S3 virus at MOI=3. The cell culture supernatant was collected at 12, and 24 h post infection. The virus titre in the supernatant was measured by the plaque assay performed in BHK cells. The bars depict mean $\pm$ SD of three independent experiments performed in duplicate.(****$p<0.0001$).

Disease Models & Mechanisms • Supplementary information

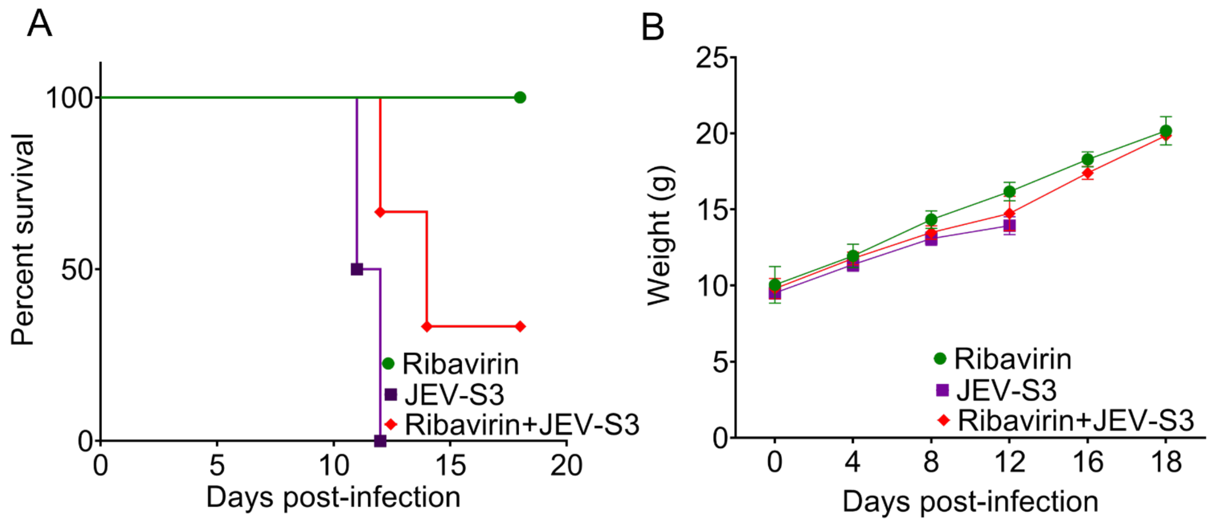

Fig. S3. Antiviral activity of Ribavirin in the JEV mouse model. C57BL/6 (3-4weeks old) mice of either sex were randomly divided into three groups (6 mice in each group). Mice in JEV-S3 and Ribavirin+JEV-S3 group were injected IP with 100 µl JEV-S3 (10^7 pfu). Ribavirin and Ribavirin+JEV-S3 groups were treated with ribavirin given IP (dose 30 mg/kg) 24 h pi with one dose each day for 7 days. The mortality was recorded. The Kaplan-Meier survival curves of mice with or without Ribavirin treatment are shown in the left panel. The body weight of mice of different experimental groups is shown in the right panel.

Disease Models & Mechanisms • Supplementary information

Disease Models & Mechanisms • Supplementary information

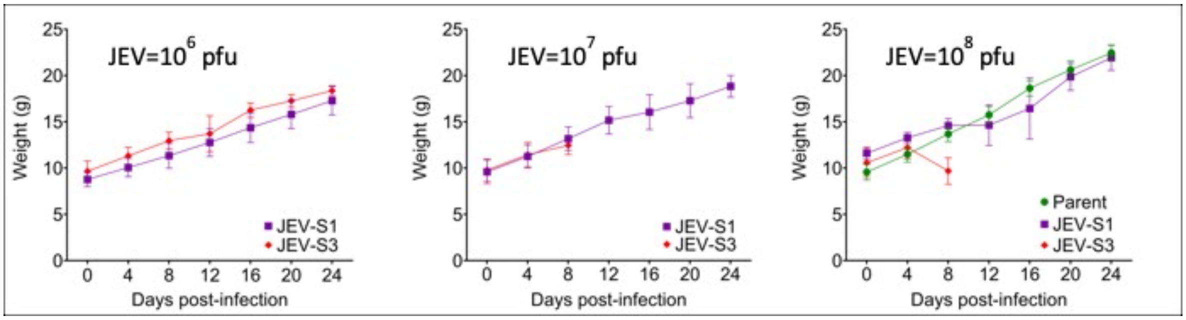

Fig. S4. Body weights of mice infected with different doses of virus. C57BL/6 mice (3-4 weeks) were inoculated IP with 100 µl JEV-S1 or S3 (10^8–10^6 pfu) (12 mice in each group) and scored for the JE clinical signs and body weight each day. The below panels show the body weight of the virus-infected animals. PI= Intraperitoneal
